# Evaluation of a Virucidal Quantitative Carrier Test for Surface Disinfectants

**DOI:** 10.1371/journal.pone.0086128

**Published:** 2014-01-27

**Authors:** Holger F. Rabenau, Jochen Steinmann, Ingrid Rapp, Ingeborg Schwebke, Maren Eggers

**Affiliations:** 1 Institute of Medical Virology, Hospital of the Johann Wolfgang Goethe University of Frankfurt, Frankfurt, Germany; 2 MikroLab GmbH, Bremen, Germany; 3 Labor Dr. Merk & Kollegen, Ochsenhausen, Germany; 4 Robert Koch-Institute, Berlin, Germany; 5 Labor Prof. G. Enders & Kollegen MVZ and Institute of Virology, Infectious Diseases and Epidemiology e.V., Stuttgart, Germany; University of Akron, United States of America

## Abstract

Surface disinfectants are part of broader preventive strategies preventing the transmission of bacteria, fungi and viruses in medical institutions. To evaluate their virucidal efficacy, these products must be tested with appropriate model viruses with different physico-chemical properties under conditions representing practical application in hospitals.

The aim of this study was to evaluate a quantitative carrier assay. Furthermore, different putative model viruses like adenovirus type 5 (AdV-5) and different animal parvoviruses were evaluated with respect to their tenacity and practicability in laboratory handling. To evaluate the robustness of the method, some of the viruses were tested in parallel in different laboratories in a multi-center study. Different biocides, which are common active ingredients of surface disinfectants, were used in the test. After drying on stainless steel discs as the carrier, model viruses were exposed to different concentrations of three alcohols, peracetic acid (PAA) or glutaraldehyde (GDA), with a fixed exposure time of 5 minutes. Residual virus was determined after treatment by endpoint titration.

All parvoviruses exhibited a similar stability with respect to GDA, while AdV-5 was more susceptible. For PAA, the porcine parvovirus was more sensitive than the other parvoviruses, and again, AdV-5 presented a higher susceptibility than the parvoviruses. All parvoviruses were resistant to alcohols, while AdV-5 was only stable when treated with 2-propanol. The analysis of the results of the multi-center study showed a high reproducibility of this test system.

In conclusion, two viruses with different physico-chemical properties can be recommended as appropriate model viruses for the evaluation of the virucidal efficacy of surface disinfectants: AdV-5, which has a high clinical impact, and murine parvovirus (MVM) with the highest practicability among the parvoviruses tested.

## Introduction

Nosocomial virus infections can prove fatal to people who are particularly vulnerable to infections. This population includes premature infants, people with chronic or degenerative illnesses, immuno-compromised patients and the elderly. In these groups, viral gastroenteritis due to rotaviruses or noroviruses, which are normally self-limiting infections, represent a harmful risk [1], [2], [3]. The same applies to respiratory viruses such as influenza, parainfluenza, enteroviruses or respiratory syncytial virus (RSV), as was recently demonstrated by a large RSV outbreak in an adult hematology unit in Heidelberg [4], [5], [6], [7]. In particular, non-enveloped viruses may persist on surfaces for several days or even months [8] and can be transferred directly from contaminated surfaces to susceptible patients [9], [10]. Therefore, the disinfection of surfaces frequently touched by patients and staff such as door handles, faucets, and railings plays an important role in the prevention and control of viral outbreaks in healthcare settings.

Considering that non-enveloped viruses such as noroviruses and enteroviruses are resistant to the majority of chemical disinfectants, only biocides with proven virucidal efficacy can be used. This can only be achieved by ensuring that disinfectants pass a virucidal activity test performed in compliance with good laboratory practice and country-specific standards. In Europe, EN 14476 describes the standard for determining virucidal activity, which involves two non-enveloped viruses and a quantitative suspension assay [11]. This quantitative suspension test is performed in a test tube. The shortcoming of this assay is that the virus particles are suspended in a large volume of disinfectant, which makes inactivation of viruses easier due to the high amount of contact between the disinfectant and virus particles.

Therefore, suspension tests do not reflect real conditions. In practice, viruses are immobilized on objects and/or work surfaces with a high protein load from body fluids, which may protect viruses from disinfection. To ensure that surface disinfectants are able to inactivate microorganisms, they must be tested for their efficacy under close to real-life conditions. This type of practical assay has long been standardized for the testing of the bactericidal activity of chemical disinfectants [12], [13], [14]. Bactericidal disinfectants are tested stepwise according to European test principles, beginning with a suspension test (EN phase 2, step 1) and then a quantitative non-porous carrier test simulating practical conditions (EN phase 2, step 2) [14], [15]. In contrast to the test for bactericidal activity, until now, no European standard for virucidal efficacy testing that simulates practical conditions exists. Such a test should be based on model viruses that can be dried on carriers. The choice of model viruses is of great importance when establishing such a carrier test. The requirements for model viruses are as follows: high resistance to disinfectants and drying, combined with simple virus propagation in cell culture. The model viruses that are already being used in the suspension test and that fulfill these requirements can be taken into account. In the suspension test, according to EN 14476 (phase 2/step 1), poliovirus type 1 LSc-2ab and adenovirus type 5 strain (AdV-5) Adenoid 75 are used as model viruses. Both viruses are suitable with respect to their resistance to active ingredients, but polioviruses have some drawbacks. In general, the use of poliovirus is only temporary, and in the future, such use will require higher biosafety levels because of the global polio eradication program, which was initiated by the World Health Organization (WHO) in 1988 [16]. Furthermore, poliovirus infectivity is significantly decreased after drying [17].

Therefore, poliovirus should be replaced with an alternative model virus. For this purpose, animal parvoviruses such as the bovine parvovirus (BPV) strain Haden, which is used for chemothermal disinfection in the quantitative suspension test and a national surface test [11], [18], [19], can be used because of their known environmental stability and their practicability for laboratory use and because they do not pose any hazard to employees performing the tests. However, BPV is not ideal because this single-stranded DNA virus requires primary cells for replication, meaning that it is difficult to handle in a routine testing laboratory. In contrast, other animal parvoviruses, such as minute virus of mice (MVM), porcine parvovirus (PPV), and canine parvovirus (CPV), are cultivable on continuously growing cells and are comparable to bovine parvovirus in terms of stability. Thus, they can be chosen as model viruses for a carrier test.

### Objectives

Our study was designed to address two objectives, which are both important for efficacy testing of surface disinfectants. The first aim was to establish a virucidal quantitative carrier test that simulates practical conditions in a similar manner to the long-established bactericidal carrier test. Viruses were dried on stainless steel discs and exposed to different biocides, which are common ingredients of commercial surface disinfectants.

The second aim was to choose and evaluate reliable model viruses for such a test using AdV-5 and different animal parvoviruses. MVM strain Crawford, PPV strain NADL-2 and CPV type 2 were compared with BPV, which is already used to test chemo-thermal inactivation [11], [18]. For murine and porcine parvoviruses, the investigation was performed as a multi-center study in order to evaluate the inter-laboratory robustness of this method.

## Materials and Methods

### Viruses and cell cultures

Bovine, canine, murine and porcine parvovirus (PV) and AdV-5 were used in the experiments. Test virus suspensions were prepared by infecting susceptible cells with different multiplicities of infection (MOI). For BPV (strain Haden, provided by Prof. Böhm, University of Hohenheim, Stuttgart, Germany), fibroblastic bovine embryonic lung (BEL) cells were used (established and propagated by Labor Prof. Enders, MOI 1); for canine PV type 2 (CPV) (kindly provided by Prof. Truyen,University of Leipzig, Leipzig, Germany), CRFK cells were used (provided by Dr. Riebe of the Collection of Cell Lines in Veterinary Medicine (CCLV), Friedrich-Loeffler-Institute, Isle of Riems, Germany, MOI 0.1); for murine PV (MVM [minute virus of mice], strain Crawford, ATCC VR-1346), A9 cells were used (European Cell Culture Collection (EACC No 85011426) provided by Paul-Ehrlich-Institute, Langen, Germany, MOI 1.0); for porcine PV (PPV; strain NADL-2, ATCC VR-742), PK13 cells were used (ATCC CRL 6489 provided by Paul-Ehrlich-Institute, MOI 1); and for AdV-5 (strain Adenoid 75, ATCC VR-5), A549 cells (ATCC CCL-185, MOI 0.01) were used.

### Isolation and culture of primary bovine embryonic lung fibroblasts

Bovine embryonic lung cell cultures were prepared from a 5-month-old calf fetus. In brief, the lung was cut into tiny pieces. The cut tissue was transfered into sterile 100 mL beaker with sterile stir bar and floated on 700 ml trypsine/EDTA solution (Biochrome, Berlin, Germany), stirring slowly for 90 min. The solution containing tissue fragments was transferred to sterile 50 mL tubes and media was added. The 50 mL tubes were spin at 1500 rpm for 10 min. The supernatant was removed and the pellet washed twice with 10 mL of MEM media with 10% FBS. Then the pellet was resuspended in 20 mL of DMEM media with 20% FBS and 1% antibiotic/antimycotic and transfered to a T75 tissue culture flask (Greiner Bio-one, Frickenhausen, Germany). The primary cells were incubated at 37°C, 5%CO2 and checked every day for fibroblasts and media colour. The trypsine procedure was repeatedly performed with the remaining lung tissue. After 7 days, fibroblasts of 3 different harvests exists and were stored in liquid nitrogen until used.

### Virus propagation

After virus inoculation of the cells, the supernatant was replaced by a suitable cell culture medium: for PPV, Iscove's Modified Dulbecco's Medium (IMEM), Biochrom AG, Germany; for MVM, PPV and BPV, Dulbecco's minimum essential medium (DMEM, Sigma-Aldrich, Germany); for AdV-5, minimum essential medium (MEM, Biochrom AG, Germany) with 10% (for MVM), 5% (for PPV) or 1% (for adenovirus) fetal calf serum (FCS, Sigma-Aldrich, Germany). The cells were incubated at 37°C until 70–95% of the cells exhibited a cytopathic effect (for adenovirus, approximately 10–12 days; for parvoviruses, 10–14 days). The cells were frozen and thawed twice, followed by centrifugation at 1600 *g* for 10 minutes. The supernatant was aliquoted as test virus suspensions and stored at −80°C.

### Biocides

Five biocides were used in this study: glutaraldehyde (GDA) (2500, 2000, 1000, 500, 125 ppm), peracetic acid (PAA) (1500, 1000, 500, 200, 50 ppm), ethanol (60, 55, 50, 45, 40%, v/v), 1-propanol (60, 50, 40, 30, 20, 10%, v/v) and 2-propanol (60, 50, 40, 30, 20%, v/v).

Dilutions of PAA, GDA and the alcohols (Sigma-Aldrich, Seelze, Germany) were prepared with hard water (300 ppm CaCO3, pH 7.0 - in accordance with EN 14476) immediately before the inactivation experiments [11].

### Preparation of virus inoculum

Nine volumes of test virus suspension were mixed with one volume of 0.3% w/v of bovine serum albumin (BSA, clean conditions), resulting in a final BSA concentration of 0.03% in the virus inoculum.

### Disc washing

The cleaning of the stainless steel discs (20 mm diameter, GK Formblech GmbH, Berlin, Germany) was performed as already described [20], [21]. In brief, prior to use, the discs were placed in a container with an appropriate quantity of 5% (V/V) Decon 90 for 60 min, in a manner that the discs don't stick together. Subsequently, the discs were rinsed with running freshly distilled water for 10 seconds. The rinsing was repeated with double distilled water for a further 10 seconds in order to ensure complete removal of the surfactant. Then, the the discs were diped in a bath containing 70% (V/V) ethanol for 15 min. At the end, the discs were removed and rinsed with double distilled water for at least 10 seconds. Sterilization was done by autoclaving.

### Preparation of the carriers and test performance

A total of 50 µl of the virus inoculum was deposited on each pre-treated carrier and dried in a desiccator (700–800 mbar, for 30 min). Then, the discs were transferred into plastic vial holders (Sarstedt AG & Co. KG, Nümbrecht, Germany) containing 0.5 g glass beads (0.25–0.50 mm diameter, Carl Roth GmbH, Karlsruhe, Germany), covered with 100 µl of the biocide (for the control carriers t_5 min_, 100 µl of hard water was applied) and incubated for 5 minutes. Immediately at the end of the exposure time, 900 µl of ice-cold culture medium was added to stop the activity of the biocides. Vials were vortexed for 1 min to recover the residual viruses, and the eluate was immediately diluted 10-fold for determining viral infectivity. For the recovery rate, 50 µl of the virus inoculum was deposited on each pre-treated carrier and was analyzed without drying (inocculum control). The t_0 min_ control was immediately determined after drying. All tests were carried out at ambient temperatures of 20–22°C with three replicates (carriers) and a minimum of two independent experiments conducted on different days.

### Determination of the infectivity and statistical analysis

Infectivity was determined by transferring 100 µl of each dilution into eight wells of a 96-well microtiter plate with permissive cells. The cell cultures were monitored for cytotoxic effects for the same incubation time as described for the virus propagation.

Virus titers were determined using the methods of Spearman [22] and Kaerber [23] and expressed as log_10_TCID_50_/ml, including standard deviation. The control titer of the different viruses ranged from 5.6×10^6^ to 3.7×10^8^ TCID_50_/ml (in detail: the titer ranges for AdV-5 were from 0.2×10^7^ to 3.7×10^8^ TCID_50_/ml; for BPV, from 0.2×10^7^ to 0.8×10^8^; for CPV, from 0.3×10^6^ to 0.4×10^6^; for MVM, from 5.6×10^5^ to 2.5×10^8^; and for PPV, from 6.3×10^5^ to 8.3×10^6^). Titer reduction is presented as the difference between the virus titer of the water control and the test sample exposed to the biocide. This difference is given as the log_10_ reduction factor (log_10_ RF), including its 95% confidence interval (CI) [18]. A 4-log_10_ reduction is required for efficacy (an inactivation of 99.99%). Biologically relevant log_10_ RF differences between viruses or laboratories were defined as ≥1 step considering the lower and upper bounds of the 95% CI [24]. Significant differences were calculated from at least 6 parallel titrations.

### Participants

Five German laboratories participated in this study – referred to as Lab 1, Lab 2, Lab 3, Lab 4, and Lab 5. Participation was open and free of charge to all laboratories.

## Results

The concentrations of the biocides used here were chosen to allow us to observe kinetics and the transition from non-efficient to efficient virus inactivation. The participating laboratories tested all biocide concentrations on 2 test days and performed the test on triplicate carriers per test day. The difference in the virus titer before and after drying on the carriers was <0.5 log_10_ (Tab. 1). However, because each laboratory used its own virus stock, the different titers of the test virus suspension from the different labs are, to some extent, responsible for any inter-laboratory and inter-virus log_10_ RF differences (Tab. 1 and Tab. 2).

**Table 1:** Concentration-dependent virucidal activity of 5 biocides against Ad-5.

		adenovirus type 5					
biocide		Lab 1		Lab 2		Lab 5	
		log_10_ TCID_50_/ml	log_10_ RF ±95%CI	log_10_ TCID_50_/ml	log_10_ RF ±95%CI	log_10_ TCID_50_/ml	log_10_ RF±95%CI
**control**	**inocculum**	**8.05**		**7.26**		n.t.	
	**t_0 min_**	n.t.		**6.61**		n.t.	
	**t_5 min_**	**8.14**		**6.42**		**6.70**	
**GDA (ppm)**	125	5.45	2.69±0.50	1.57	4.85±0.45	2.18	4.52±0.50
	500	2.36	5.77±1.23	1.50	4.92±0.44	1.50	5.20±0.54
	1000	1.86	6.27±0.35	1.50	4.92±0.44	1.50	5.20±0.54
	2000	1.86	6.27±0.35	2.50	3.92±0.44	1.50	5.20±0.54
	2500	1.80	6.34±0.32	2.50	3.92±0.44	1.50	5.20±0.53
**control**	**inocculum**	n.t.		**7.26**		n.t.	
	**t_0 min_**	n.t.		**6.61**		n.t.	
	**t_5 min_**	**7.63**		**6.42**		**7.44**	
**PAA (ppm)**	50	7.65	−0.02±0.38	6.44	−0.02±0.59	7.17	0.33±0.54
	200	7.21	0.41±0.58	1.84	4.58±0.68	4.02	2.92±0.52
	500	4.82	2.81±0.96	1.50	4.92±0.44	1.99	5.45±0.53
	1000	2.50	5.13±0.25	1.50	4.92±0.44	1.67	5.77±0.45
	1500	2.52	5.11±0.26	1.50	4.92±0.44	1.50	5.94±0.36
**control**	**inocculum**	n.t.		**7.13**		n.t.	
	**t_0 min_**	n.t.		**6.50**		n.t.	
	**t_5 min_**	**8.18**		**6.67**		**7.73**	
**ethanol (v/v)**	40%	7.95	0.23±0.90	5.98	0.69±0.39	3.64	2.55±0.52
	45%	6.80	1.38±0.79	n.t.	n.t.	n.t.	n.t.
	50%	6.28	1.90±1.39	5.04	1.63±0.55	1.50	6.23±0.41
	55%	3.26	4.92±1.11	1.54	5.13±0.31	1.50	6.23±0.41
	60%	2.68	5.50±0.55	1.50	5.17±0.30	1.50	6.23±0.41
**control**	**inocculum**	n.t.		**7.38**		n.t.	
	**t_0 min_**	n.t.		**6.77**		n.t.	
	**t_5 min_**	**8.32**		**6.65**		**7.21**	
**1-pro-panol (v/v)**	10%	8.18	0.15±0.00	5.69	0.96±0.45	6.93	0.28±0.54
	20%	7.72	0.60±0.39	5.52	1.13±0.91	1.55	5.66±0.41
	30%	3.05	5.27±0.87	1.54	5.11±0.39	1.50	5.71±0.40
	40%	2.70	5.63±0.51	1.67	4.98±0.43	1.50	5.71±0.40
	50%	2.39	5.94±0.62	1.61	5.04±0.47	1.50	5.71±0.40
	60%	3.80	4.52±0.75	1.90	4.75±0.45	1.50	5.71±0.40
**control**	**inocculum**	n.t.		**7.38**		n.t.	
	**t_0 min_**	n.t.		**6.77**		n.t.	
	**t_5 min_**	**8.34**		**6.65**		n.t.	
**2-pro-panol (v/v)**	20%	7.97	0.37±0.27	n.t.	n.t.	n.t.	n.t.
	30%	7.68	0.67±0.50	n.t.	n.t.	n.t.	n.t.
	40%	7.43	0.92±0.36	6.08	0.57±0.39	n.t.	n.t.
	50%	7.01	1.33±0.40	n.t.	n.t.	n.t.	n.t.
	60%	7.10	1.25±0.92	5.90	0.75±0.39	n.t.	n.t.

**Table 2:** Concentration-dependent virucidal activity of 5 biocides against animal parvoviruses.

		minute virus of mice				porcine parvovirus						bovine parvovirus		canine parvovirus	
		Lab 3		Lab 4		Lab 1		Lab 3		Lab 5		Lab 4		Lab 2	
		log_10_ TCID_50_/ml	log_10_ RF ±95%CI	log_10_ TCID_50_/ml	log_10_ RF ±95%CI	log_10_ TCID_50_/ml	log_10_ RF ±95%CI	log_10_ TCID_50_/ml	log_10_ RF±95%CI	log_10_ TCID_50_/ml	log10 RF±95%CI	log_10_ TCID_50_/ml	log_10_ RF±95%CI	log_10_ TCID_50_/ml	log_10_ RF±95%CI
Control	**inocculum**	**5.80**		**8.03**		n.t.		**5.42**		n.t.					
	**t_0 min_**	**5.54**		**8.52**		n.t.		**5.71**		n.t.					
	**t_5 min_**	**5.53**		**8.40**		**7.03**		**5.82**							
GDA (ppm)	125	4.88	0.65±0.34	7.96	0.44±0.38	6.57	0.46±0.36	5.38	0.43±0.23		0.48±0.65		0.49±0.41		0.52±0.55
	500	2.93	2.61±0.67	6.88	1.52±0.38	5.87	1.17±0.39	3.91	1.92±0.26		1.31±0.57		2.09±0.35		2.52±0.58
	1000	2.19	3.34±1.02	5.90	2.50±0.38	5.22	1.81±0.29	3.00	2.82±0.75		1.73±1.11		2.71±0.39		2.98±0.58
	2000	1.52	4.01±0.27	5.15	3.25±0.35	3.91	3.13±0.31	1.98	3.84±0.43		3.32±0.64		2.88±0.36		3.05±0.53
	2500	1.50	4.03±0.26	4.09	4.31±0.36	3.10	3.94±0.38	1.95	3.87±0.58		3.65±0.79		3.61±0.35		3.05±0.53
control	**inocculum**	**6.25**		**8.44**		n.t.		**6.7**		n.t.		**7.06**		**5.66**	
	**t_0 min_**	**5.81**		**8.57**		n.t.		**6.17**		n.t.		**7.32**		**5.61**	
	**t_5 min_**	**6.00**		**8.56**		**6.86**		**6.11**		**7.11**		**7.55**		**5.55**	
PAA (ppm)	50	5.77	0.23±0.41	8.25	0.31±0.35	6.23	0.63±0.63	5.12	0.99±0.35	6.46	0.65±1.06	6.84	0.71±0.37	5.50	0.04±0.59
	200	4.90	1.10±0.32	7.79	0.77±0.35	5.48	1.38±0.50	2.41	3.70±0.78	3.21	3.90±1.00	5.80	1.75±0.40	5.11	0.44±0.59
	500	3.92	2.08±0.37	6.90	1.67±0.35	4.73	2.13±0.37	2.10	4.01±1.04	1.67	5.44±1.02	5.56	1.99±0.37	4.78	0.77±0.65
	1000	3.07	2.94±0.27	6.32	2.25±0.35	3.11	3.75±0.57	1.79	4.32±0.69	1.61	5.50±0.98	4.98	2.57±0.34	3.90	1.65±0.59
	1500	2.69	3.31±0.39	6.00	2.56±0.34	2.59	4.27±0.34	1.76	4.35±0.55	1.50	5.61±0.97	4.84	2.71±0.39	3.57	1.98±0.60
control	**inocculum**	**5.80**		**8.25**		n.t.		**5.42**		n.t.		**7.40**		**5.75**	
	**t_0 min_**	**5.50**		**8.42**		n.t.		**5.57**		n.t.		**7.69**		**5.71**	
	**t_5 min_**	**5.88**		**8.38**		**6.95**		**5.72**		**5.90**		**7.65**		**5.63**	
**ethanol (v/v)**	10%	5.36	0.52±0.97	8.52	−0.14±0.38	6.51	0.44±0.24	5.88	−0.16±0.40	5.85	0.05±0.28	7.04	0.61±0.39	5.36	0.27±0.51
	20%	5.64	0.24±0.98	8.42	−0.04±0.38	6.39	0.56±0.33	5.69	0.03±0.29	5.77	0.13±0.27	6.84	0.81±0.39	n.t.	n.t.
	30%	5.60	0.28±0.72	8.32	0.06±0.37	6.03	0.91±0.37	5.50	0.22±0.37	5.77	0.13±0.34	6.82	0.83±0.41	5.65	−0.02±0.49
	40%	5.41	0.47±0.86	8.29	0.08±0.37	6.20	0.75±0.41	5.50	0.22±0.37	5.62	0.28±0.31	6.71	0.94±0.38	n.t.	n.t.
	50%	5.36	0.52±0.64	8.27	0.10±0.36	6.05	0.90±0.26	4.69	1.03±0.50	5.32	0.58±0.27	6.69	0.96±0.40	5.21	0.42±0.47
control		**5.80**		**8.25**		n.t.		**5.42**		n.t.		**7.40**		**5.80**	
		**5.79**		**8.42**		n.t.		**5.64**		n.t.		**7.69**		**5.79**	
		**5.79**		**8.38**		**6.76**		**6.07**		**5.90**		**7.65**		**5.79**	
**1-pro-panol (v/v)**	10%	5.88	−0.09±1.31	8.56	−0.18±0.32	n.t.	n.t.	n.t.		5.63	0.27±0.17	7.19	0.46±0.39	5.88	−0.09±1.31
	20%	6.12	−0.33±1.34	8.42	−0.04±0.34	6.66	0.10±0.25	5.60	0.47±0.66	5.72	0.18±0.41	7.05	0.60±0.40	6.12	−0.33±1.34
	30%	5.79	0.00±0.65	8.34	0.04±0.36	6.47	0.29±0.20	5.98	0.09±0.82	5.77	0.13±0.36	6.98	0.67±0.40	5.79	0.00±0.65
	40%	5.79	0.00±0.65	8.29	0.09±0.36	6.41	0.35±0.16	5.41	0.66±0.52	5.60	0.30±0.41	6.92	0.73±0.38	5.79	0.00±0.65
	50%	5.45	0.33±0.67	8.25	0.13±0.35	6.41	0.35±0.22	5.69	0.38±0.66	5.88	0.02±0.57	6.82	0.83±0.41	5.45	0.33±0.67
	60%	5.55	0.24±1.28	8.13	0.25±0.32	6.45	0.31±0.24	5.31	0.76±0.66	5.21	0.35±0.55	6.75	0.90±0.37	5.55	0.24±1.28
control		**5.80**		**8.25**		n.t.		**5.42**		n.t.		**7.40**		**5.75**	
		**5.50**		**8.42**		n.t.		**6.15**		n.t.		**7.69**		**5.71**	
		**5.88**		**8.38**		**6.99**		**5.86**		**5.60**		**7.65**		**5.63**	
**2-pro-panol (v/v)**	20%	5.64	0.24±0.96	8.67	−0.29±0.35	6.43	0.56±0.41	5.98	−0.12±0.68	5.52	0.08±0.12	7.29	0.36±0.41	n.t.	n.t.
	30%	6.02	−0.14±1.17	8.52	−0.14±0.35	6.49	0.50±0.30	5.93	−0.07±0.83	5.38	0.22±0.31	7.23	0.42±0.42	n.t.	n.t.
	40%	5.98	−0.10±1.19	8.40	−0.02±0.36	6.32	0.67±0.27	5.83	0.03±0.74	5.40	0.20±0.26	7.17	0.48±0.43	5.46	0.17±0.56
	50%	5.52	0.36±0.72	8.29	0.09±0.34	6.22	0.77±0.29	5.45	0.41±0.74	5.33	0.27±0.22	7.02	0.63±0.41	5.65	−0.02±0.52
	60%	5.38	0.50±0.86	8.03	0.35±0.33	6.30	0.69±0.24	5.50	0.36±0.66	5.47	0.13±0.18	6.92	0.73±0.40	5.25	0.38±0.53

For ethanol and 1-propanol, virucidal efficacy was observed only for AdV-5 (Tab. 1), and all parvoviruses were not sufficiently inactivated (Tab. 2). At concentrations ≥55% (v/v) for ethanol and ≥30% (v/v) for 1-propanol, the titer reduction for AdV-5 was at least 4 log_10_ steps (Tab. 1). Only low inactivation was observed with 2-propanol.

**Figure 1:**
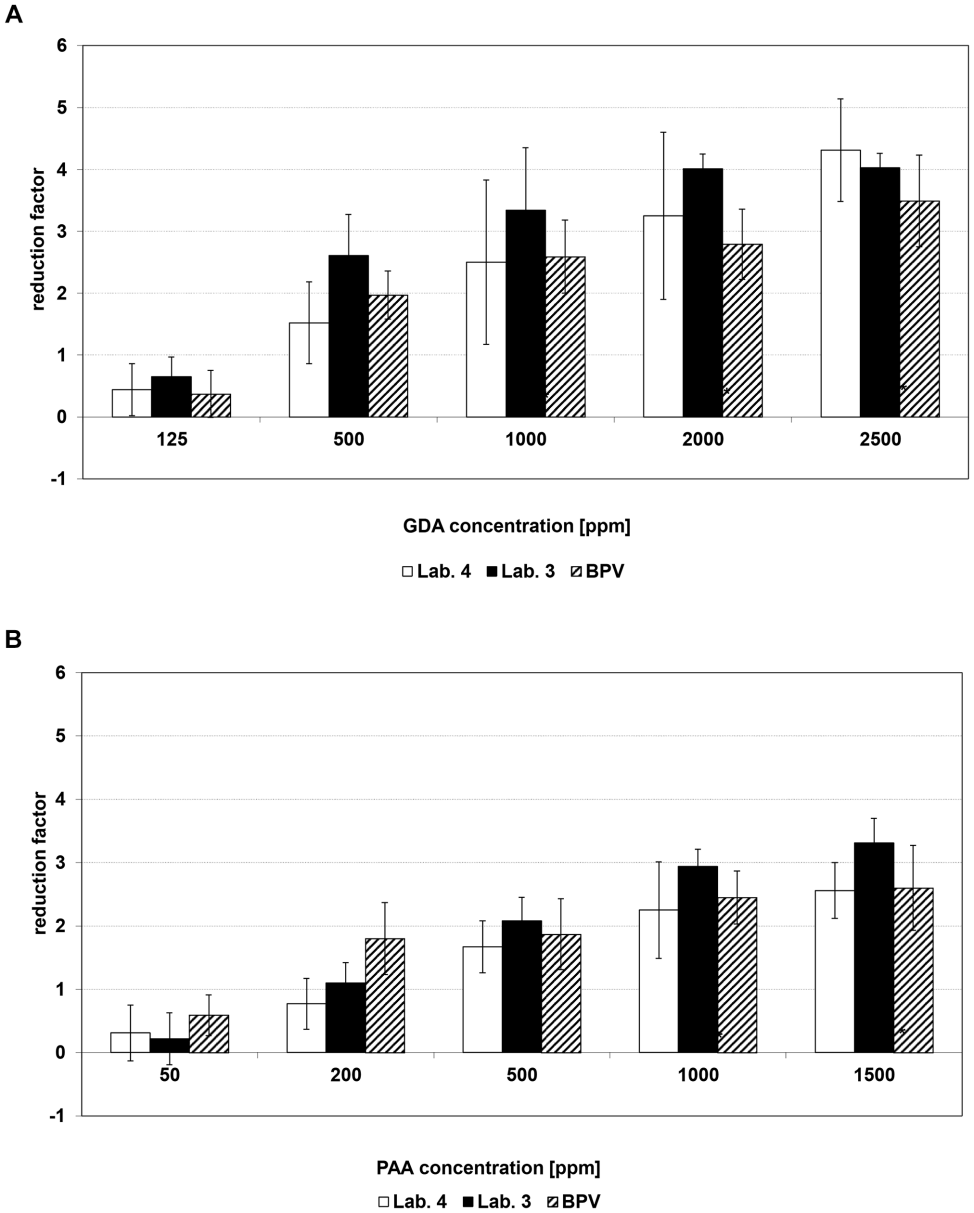
Concentration-dependent virucidal efficacy of a) glutaraldehyde and b) peracetic acid against MVM tested in 2 of the 5 laboratories compared to BPV (vertical lines indicate the 95% confidence intervals).

**Figure 2:**
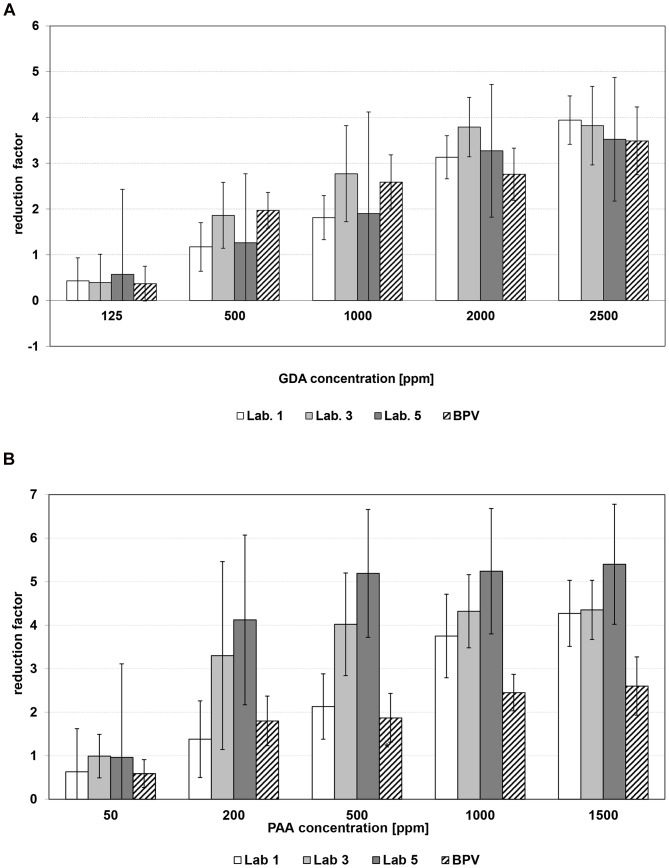
Concentration-dependent virucidal efficacy of a) glutaraldehyde and b) peracetic acid against PPV tested in 3 of the 5 laboratories compared to BPV (vertical lines indicate the 95% confidence intervals).

A ≥4 log_10_ inactivation of AdV-5 was also detected for GDA at concentrations of 125 to 500 ppm (Tab. 1). Furthermore, a concentration-dependent increase of the virucidal efficacy of GDA was detectable for all parvoviruses. GDA at 500 ppm was not able to sufficiently reduce parvovirus titers (Tab. 2). At concentrations of 2500 ppm, a 4 log_10_ reduction of only MVM was detected, although residual virus could still be found (Fig. 1 a). Whereas PPV showed similar kinetic as BPV (Fig. 2 a). Testing CPV, a 99.99% reduction was limited by the cytotoxicity and/or the titer of the virus inoculum used (Tab. 2). Therefore, in some cases, the log_10_ RFs are marked with “≥”, indicating that the determination of the log_10_ RF was limited due to cytotoxicity.

With respect to PAA, a concentration-dependent log_10_ RF increase could be observed for all parvoviruses (Tab. 2). MVM was as resistant to PAA as BPV (Fig. 1 b), while PPV was more susceptible to PAA (Fig. 2 b). For AdV-5, an log_10_ RF ≥4 was detected at PAA concentrations ≤1000 ppm (Tab. 1).

In summary, our data reveal a concentration-dependent virucidal efficacy for the biocides. All parvoviruses have a similar level of stability when treated with GDA, while PPV is more sensitive to PAA than the others. Additionally, AdV-5 exhibited higher susceptibility when compared to the parvoviruses.

For the five biocides, all log_10_ RF data were compared with regard to relevant intra- and inter-laboratory differences of the tested viruses. No such biologically relevant differences were observed for MVM and PPV when treated with GDA and PAA. In one case, the inactivation kinetics of PPV resulted in an inter-laboratory heterogeneous (non-relevant) discrepancy between two labs (500 ppm PAA). Furthermore, no intra-laboratory differences were demonstrated for all tested viruses (Tab. 1 and 2).

## Discussion

Although surface disinfection with virucidal products is an important tool in the prevention of nosocomial viral infections, information on the efficacy of virucidal disinfectants is still missing. Disinfection research in virology is somewhat neglected, although prevalence studies and personnel monitoring have shown contamination hazards [25]. The useful dilutions and contact time of virucidal surface disinfectants are still based on concentrations that will pass either an European suspension test, that is, EN 14476, or a national standard, such as the German DVV/RKI suspension test [11], [18]. In order to improve time/concentration relations of surface disinfectants which reflect dayly needs in clinical surroundings further more practical testing is necessary. Therefore, a virucidal quantitative carrier test that simulates practical conditions was evaluated in this study. The method was based on the EN 13697 standard for the following reasons [14], [20]: In 1993, experts from the veterinary, food, industrial, domestic and institutional fields within the framework of CEN/TC 216 developed such a quantitative surface test for bactericidal and fungicidal products that mimics real conditions [26]. Derived from this work is the European standard EN 13697 [14], a quantitative non-porous surface test for the evaluation of the bactericidal and/or fungicidal activity of chemical disinfectants without mechanical action (phase 2, step 2). Ring trials during the 1990s demonstrated that this surface test, when using dried bacteria or fungi on stainless steel carriers, could result in 10-fold lower reduction factors than those obtained using suspension tests [13]. This observations are also supported by studies of Peters and Spicher on *Staphylococcus aureus* which showed an increased resistance to formaldehyde, from 0.8% in the suspension test to 1.2% in the surface test [27], [28]. Based on this observatiuons, it is assumed that virucidal disinfectants might also show an increased resistance in the surface test. However, a virucidal surface test is required to substantiate this notion and for further knowledge.

The next issue of this study was to find suitable candidates for the test viruses of a surface test.. In Europe, model viruses that cover a broad spectrum of physico-chemical properties have been used for testing and verifying the efficacy of disinfectants under practical conditions. Currently, poliovirus and AdV-5 are used by the European Standard EN 14476 as model viruses for quantitative suspension tests [11]. Poliovirus was excluded from this study because it is relatively sensitive to the drying process, with a loss of titer of approximately 3 log_10_ [17]. In addition, due to the WHO polio eradication program, poliovirus will need to be substituted with another model virus with similar properties in the future. The thermoresistant virus BPV is used for the determination of the virucidal activity of chemothermal disinfection procedures in both the EN 14476 and the DVV/RKI guideline. However, BPV is difficult to grow in the laboratory. In contrast, PPV and MVM are easy to propagate. Therefore, this study investigated the suitability of AdV-5 and four different animal parvoviruses (BPV, CPV, MVM, and PPV) as putative model viruses that can potentially substitute for poliovirus.

One of the basic requirements for a model virus is stability during a drying process. Our data showed that all of the viruses we tested fulfill this requirement. The difference between the virus titer before and after drying on the carriers was ≤0.5 log_10_ (data not shown). This result was to be expected because all animal parvoviruses are known to be very stable when exposed to environmental influences such as chemicals or heat [29]. In contrast, it is known that many enveloped viruses lose significant infectivity meaning a small difference between virus after drying and the limit of virus detection [30].

Furthermore, our results demonstrated that in all tests with BPV, residual viruses were confirmed. Thus, 99.99% inactivation could not be achieved using the tested conditions. Most of the tested parvoviruses revealed similar log_10_ reductions with GDA (Tab. 2). Only for PPV exposed to PAA was a 4 log_10_ reduction achieved. Similar to results were found by Eterpi et al., the parvoviruses (PPV, MVM) exhibited higher stability than the adenovirus when exposed to different active substances and disinfectants [29]. Our experiments showed also that GDA and PAA were found to be very active against AdV-5 (Tab. 1). A log_10_ reductions of ≥4 of the adenovirus was achieved by ≥250 ppm (range 125 to 500) GDA, 500 ppm (range 200 to 1000) PAA. While 1-propanol (30% v/v) was the most effective alcohol used to inactivate AdV-5 on the carrier, for ethanol, higher concentrations (≥55%) were needed. Treatment with 2-propanol did not result in a 4 log_10_ reduction of AdV-5, even at a concentration of 60% (v/v). Similar ranges for GDA, PAA, and Ethanol were fond in study carried out by the virucidal task group of CEN/TC 216: Adv-5 was inactivated in a carrier test by 500 ppm GDA, ≤500 ppm PAA, and ≥60% (v/v) Ethanol (personal communication, Dr. Graziella Morace, VTG Project Leader of TC 216). Variation in the activity range whether of different sources or passages of viruses and cell lines were also found in the VTG study. Therefore, reference test for virus inactivation with a defined product (e.g. GDA and PAA) in parallel with a product under test for internal control of the test is needed.

All PVs were stable against all tested alcohols (Tab. 2). Stability against ethanol and 2-propanol has also been demonstrated previously in tests with two parvoviruses (CPV and Kilham rat virus), confirming our data [31]. Although in this study, BPV and CPV demonstrated the highest stability, both viruses are difficult to handle in the laboratory. In contrast, MVM reaches high viral titers and is relatively uncomplicated to handle in the laboratory. Our data on MVM reveal that the test accuracy across the participating laboratories was relatively high, and the inactivation kinetic was close to that of the established test virus BPV.

Therefore, along with MVM, AdV-5, despite its more fragile character, seems to be an appropriate model virus, particularly due to its biological properties and the importance of adenoviruses in human medicine. Furthermore, due to the clinical impact of human noroviruses, we recommend the additional use of murine norovirus (MNV), a surrogate virus for human noroviruses, in combination with AdV-5 and MVM for future guidelines or standards that evaluate the virucidal efficacy of surface disinfectants. In the revised version of prEN 14476:2013, MNV is included as a test virus. In the food and industrial sectors, multiple studies evaluating norovirus inactivation on surfaces have been recently performed [21], [32], [33], [34], [35]. Some studies were carried out using MNV on stainless steel carriers [21], [35]. Magulski et al. [21] tested the inactivating properties of several chemical biocides using MNV. MNV demonstrated a similar stability during drying as AdV-5 and parvoviruses. The virucidal susceptibility of MNV is similar to those of PPV in our study [21]. Concentrations of 1500 ppm for PAA and 2500 ppm for GDA were needed to inactivate 99.99% of MNV within 5 min [21]. However, the activity range of alcohols was nearly the same as for AdV-5 in our study. Ethanol (50%, v/v) and 1-propanol (30%, v/v) could inactivate MNV-1 on stainless steel discs by 4 log_10_ units within 5 min, and 2-propanol was not active. As already mentioned, one of the aims of our study was to evaluate the reproducibility of the method used. We could show, that there were no considerable intra- and inter-laboratory log_10_ RF differences discerned for the PVs. The observed log_10_ RF discrepancies were found at concentrations with low efficacy and might be caused by a number of factors (e.g., the manner of the preparation of the test virus suspension, limitations due to cytotoxic reactions, inactivation kinetics). Our study has some limitations that are based on the nature of virus cultivation. For example, if all labs used viruses and cells from the same origin at an identical passage number and equal culture media, as well as under other identical conditions, the results would perhaps differ to a lesser extent. On the other hand, the performance of our study includes the biological variety that appears during disinfectant testing. High standardization for virus cultivation and reproducible measures to minimize cytotoxicity are necessary for performing this method. Further standardization of this method might be achieved by the introduction of certain reference biocides with defined reference ranges, such as GDA, PAA, or ethanol. This and further ring trials will contribute to improving the comparability of virucidal tests between laboratories.

Nevertheless, our results present a feasible method that enables an efficacy assessment that simulates applications in hospital settings.

## Conclusion

Based on our results MVM and AdV-5 can be recommended as appropriate model viruses for the evaluation of virucidal efficacy of surface disinfectants. These viruses, together with MNV as a surrogate of human norovirus, are well-suited model viruses for test guidelines that simulate practical conditions for surface disinfectants using stainless steel carriers as the surface. In addition, all viruses can be cultivated to high titers and demonstrate high stability during the drying process.
